# A Community-Based Physical Activity Counselling Program for People With Knee Osteoarthritis: Feasibility and Preliminary Efficacy of the Track-OA Study

**DOI:** 10.2196/mhealth.7863

**Published:** 2017-06-26

**Authors:** Linda C Li, Eric C Sayre, Hui Xie, Cam Clayton, Lynne M Feehan

**Affiliations:** ^1^ Department of Physical Therapy University of British Columbia Vancouver, BC Canada; ^2^ Arthritis Research Canada Richmond, BC Canada; ^3^ Faculty of Health Sciences Simon Fraser University Burnaby, BC Canada; ^4^ Rehabilitation Program Fraser Health Surrey, BC Canada

**Keywords:** osteoarthritis, physical activity, sedentary behavior, sedentary lifestyle, wearables, digital technology, fitness trackers, exercise

## Abstract

**Background:**

Physical activity can improve health outcomes in people with knee osteoarthritis (OA); however, participation in physical activity is very low in this population.

**Objective:**

The objective of our study was to assess the feasibility and preliminary efficacy of the use of wearables (Fitbit Flex) and telephone counselling by a physical therapist (PT) for improving physical activity in people with a physician-confirmed diagnosis of knee OA, or who have passed 2 validated criteria for early OA.

**Methods:**

We conducted a community-based feasibility randomized controlled trial. The immediate group (n=17) received a brief education session by a physical therapist, a Fitbit Flex activity tracker, and a weekly telephone call for activity counselling with the physical therapist. The delayed group (n=17) received the same intervention 1 month later. All participants were assessed at baseline (T0), and the end of 1 month (T1) and 2 months (T2). Outcomes were (1) mean moderate to vigorous physical activity time, (2) mean time spent on sedentary behavior, (3) Knee Injury and Osteoarthritis Outcome Score (KOOS), and (4) Partners in Health Scale. Feasibility data were summarized with descriptive statistics. We used analysis of covariance to evaluate the effect of the group type on the outcome measures at T1 and T2, after adjusting for blocking and T0. We assessed planned contrasts of changes in outcome measures over measurement periods.

**Results:**

We identified 46 eligible individuals; of those, 34 (74%) enrolled and no one dropped out. All but 1 participant adhered to the intervention protocol. We found a significant effect, with the immediate intervention group having improved in the moderate to vigorous physical activity time and in the Partners in Health Scale at T0 to T1 compared with the delayed intervention group. The planned contrast of the immediate intervention group at T0 to T1 versus the delayed group at T1 to T2 showed a significant effect in the sedentary time and the KOOS symptoms subscale, favoring the delayed group.

**Conclusions:**

This study demonstrated the feasibility of a behavioral intervention, supported by the use of a wearable device, to promote physical activity among people with knee OA.

**Trial Registration:**

ClinicalTrials.gov NCT02313506; https://clinicaltrials.gov/ct2/show/NCT02313506 (Archived by WebCite at http://www.webcitation.org/6r4P3Bub0)

## Introduction

It is well known that physical activity can improve pain, mobility, and quality of life in people with knee osteoarthritis (OA) [1-4]. Being physically active is important in OA management partly due to its effect in managing weight [5-7]; however, participation in physical activity is very low in this population. A 2011 study using accelerometers found that over 90% of people with knee OA did not meet the physical activity guidelines of 150 minutes of moderate to vigorous physical activity (MVPA) per week [8]. A survey of 1713 people with knee or hip OA in Canada reported that fewer than half walked “one or more hours per week for exercise,” even among people with mild symptoms [9]. The 2011 Canadian Community Health Survey also found that 57% of people with arthritis were physically inactive during their leisure time, compared with 46% of those without arthritis (Multimedia Appendix 1). These findings concur with a 2013 systematic review that found that only 13% of people with OA met physical activity guidelines [10].

The current public health message is that *being active is good*, but people with OA may have difficulties with MVPA due to pain [11-13]. In this situation, people can still benefit from maintaining a level of light activity. Studies have indicated that a sedentary lifestyle (ie, too much sitting) is a predictor of poor health outcomes [14-18]. The detrimental health effect of sitting too much is independent of the person’s activity level. Interestingly, light activities, even done below the moderate-intensity level (eg, daily tasks done while standing or walking slowly), can provide health benefits [19-21]. Hence, there is a need for interventions to both improve the time spent in MVPA and decrease sitting time.

Nowadays, wearable devices are popular in the consumer space to support an active lifestyle. Evidence suggests they may also be beneficial in clinical populations. For example, Talbot et al [22] combined a pedometer-driven walking program with self-management education for people with knee OA and found an average increase of 23% in individuals’ daily steps and of 21% in isometric quadriceps muscle strength, compared with an education only group [22]. A 2007 meta-analysis of 8 randomized controlled trials (RCTs) reported a significant difference in the improvement of physical activity among pedometer users compared with controls (mean difference 2491 steps/day, 95% CI 1098-3885) [23].

Compared with pedometers, wearable devices such as fitness bands and smart watches offer additional features, such as the ability to track the intensity of activities and to visualize activity performance over time. These features enable individuals to set specific goals, monitor progress, and obtain real-time feedback on goal attainment. Despite their popularity, the value of wearables to improve physical activity behavior has been challenged. In a review of 13 consumer wearables, Lyons et al [24] concludes that these devices usually include motivational techniques, such as self-monitoring and real-time feedback, but rarely address skills such as action planning and problem solving, which are essential to changing physical activity behavior. In a systematic review of 11 studies evaluating wearables (1272 participants), Lewis et al [25] found preliminary evidence of improvement in physical activity participation and body weight, but no difference when compared with other behavioral change interventions. Only 1 of the included studies was deemed to be of high quality. These results suggest that future research should develop better strategies to incorporate wearables in multifaceted physical activity interventions, rather than evaluating wearables as a standalone tool. Moreover, more rigorous research design should be employed in future RCTs. The purpose of our study was, therefore, to assess the feasibility of a strategy, which combines the use of wearables and telephone counselling by a physical therapist (PT), for improving physical activity behavior in people with knee OA. The results will inform the development of a community-based RCT.

## Methods

### Study Design and Participant Eligibility

The Track-OA feasibility study [26] used a randomized, delayed-control design, whereby the randomization determined the timing of when the intervention was provided (ie, immediately vs a 1-month delay). As such, preliminary efficacy could be assessed within a conventional RCT (ie, with an intervention group and a control group) at 1 month, while all participants received the intervention after 1 month. This study design is the best suited for complex interventions with components that are likely beneficial and present a low risk to participants (eg, promoting physical activity). By ensuring that all participants receive the intervention at the end of a study, this design might promote protocol compliance.

Eligible individuals were patients who had a physician-confirmed diagnosis of knee OA, or passed 2 criteria for early OA: (1) being age 50 years or older, and (2) having experienced pain or discomfort in or around the knee during the previous year lasting 28 or more separate or consecutive days. In a community-based study by Marra et al [27], 191 of 195 (98.0%) urban-dwelling participants who met these criteria also met the American College of Rheumatology clinical criteria for knee OA [28].

We excluded individuals who (1) had a diagnosis of inflammatory arthritis, connective tissue diseases, fibromyalgia, or gout, (2) had used disease-modifying antirheumatic drugs or gout medications, (3) had knee arthroplasty, (4) were on the waitlist to receive total knee arthroplasty, (5) had acute knee injury in the past 6 months, (6) did not have an email address or daily access to a personal computer with Internet access, (7) had a body mass index of 40 kg/m^2^ or more, (8) had received a steroid injection in the last 6 months, (9) had received hyaluronate injection in a knee in the last 6 months, (10) were using medications that impaired activity tolerance (such as β-blockers), or (11) had an inappropriate level of risk for increasing their unsupervised physical activity. Potential participants completed the Physical Activity Readiness Questionnaire (PAR-Q; 2014 version) [29]. If the PAR-Q identified a potential risk, we required physician confirmation to ensure that the person was able to be physically active without the supervision of a health care professional.

We recruited participants from 3 sources: (1) postings on Facebook, Twitter, Kijiji, Craigslist, and the Arthritis Research Canada website, (2) emails sent by the Arthritis Consumer Experts (Vancouver, BC, Canada), a nonprofit patient education organization, to their patient members, and (3) emails sent by the Vancouver Coastal Health Research Institute (Vancouver, BC, Canada) to its staff. Interested individuals were invited to contact the research coordinator, who provided details about the study, screened respondents for eligibility, and obtained their informed consent.

After completing the baseline assessment, participants were randomly assigned to the immediate group or the delayed group in a 1:1 allocation ratio. The delayed group received the same intervention as the immediate group after a 1-month wait. Random numbers were generated in variable block sizes for the random allocation.

### Intervention

The intervention involved participants attending a 1.5-hour session, where they received (1) a standardized group education session about physical activity, (2) a Fitbit Flex (Fitbit, Inc, San Francisco, CA, USA), and (3) individual weekly activity counselling with a PT by telephone. The education session, delivered in groups of 2 to 3 participants, addressed the benefits of physical activity, the detrimental effects of sedentary behavior, and ways to be active without aggravating OA symptoms. The counselling component followed the brief action planning approach [30], whereby the PT guided participants to identify their activity goals, develop an action plan, identify barriers and solutions, and then rate their confidence in executing the plan. The process was repeated until the confidence rating reached at least 7 out of 10, indicating that the person was confident about implementing the plan. For sedentary behavior, the PT began by asking participants to estimate their sitting time in a normal day and identify ways to break up the sitting time. They then repeated the goal setting and confidence assessment.

Participants were then provided a Fitbit to be worn at the wrist of the nondominant side to track their physical activity behavior. They were instructed to wear the fitness band 24 hours a day except during water-based activity or when charging the device. The data were wirelessly synchronized with Fitbit’s online dashboard that could be viewed only by the participants and their study PTs. During the intervention period, the PT reviewed each individual’s physical activity on the dashboard and progressively modified the activity goals during 4 weekly 20-minute telephone calls. Participants could also contact the PT via email. At the end of the intervention, they could keep the Fitbit, but no longer had access to the PT.

### Feasibility Assessment

Guided by Bowen et al [31] and Thabane et al [32], the feasibility assessment focused on *implementation*, *practicality*, and *preliminary efficacy*. We measured implementation by the recruitment rate, dropout rate, adherence to the study protocol, and equipment retention. We aimed to achieve the following: (1) at least 80% of eligible individuals agreeing to participate, (2) no more than 10% dropping out, (3) at least 85% adhering to the intervention and assessment protocol, and (4) no more than 10% loss or malfunction of the 20 SenseWear accelerometer devices (BodyMedia, Pittsburgh, PA, USA) used in the study (for measuring the primary outcome; see below). We assessed practicality by self-reported adverse events and adherence to the assessment protocol. Specifically, participants were required to wear a SenseWear armband monitor for at least 20 hours during at least 4 of the 7 days of each evaluation period [33] and to complete all questionnaires within 7 days of the scheduled date. We assessed preliminary efficacy by examining outcome measures at baseline, and at the end of months 1 and 2.

### Outcome Measures

The primary outcome was mean time spent in bouted MVPA per day. We defined a bout as at least 10 consecutive minutes at the level of at least 3 metabolic equivalent tasks (METs; ie, the lower bound of MVPA), with allowance for interruption of up to 2 minutes below the threshold [34]. Participants received a SenseWear Mini armband sensor by courier and wore it 24 hours a day for 7 consecutive days, with the exception of removal for water-based activities. Unlike Fitbit, which is a commercial activity tracker with important limitations in measurement accuracy [35], SenseWear is a research-based accelerometer and sensor with established measurement properties [36]. Tierney et al [37] showed that SenseWear is a valid tool for estimating energy expenditure during daily activities in people with arthritis (intraclass correlation coefficient 0.72). Additional analysis was performed with a cutoff at 4 or more METs, which reflects an activity level of brisk or faster walking (ie, purposeful activities) [38].

Secondary outcomes were the mean time spent in sedentary behavior, the Knee Injury and Osteoarthritis Outcome Score (KOOS) [39,40], and the Partners in Health Scale [41]. Compared with Fitbit, SenseWear is a superior outcome measure because of its ability to differentiate between sedentary and light activities [36]. We calculated the mean daily time spent with an energy expenditure of at least 1.5 METs, occurring in bouts of more than 20 minutes during waking hours [18,21,42,43]. The KOOS consists of 5 subscales: Pain, Symptoms, Activity of Daily Living, Sports and Recreation Function, and Knee-related Quality of Life. It was originally developed for people recovering from anterior cruciate ligament and meniscus injury and has been validated in people with OA [39,40]. The Partners in Health Scale is a 12-item measure designed to assess perceived self-management capacity via subjective knowledge of the health condition and treatment, and perceived self-management behavior (eg, adopting a healthy lifestyle) (Cronbach alpha=.82) [41]. We also tracked self-reported adverse events (falls, cardiovascular and musculoskeletal events) [44] using a monthly log completed by the participants.

**Table 1:** Baseline characteristics of immediate intervention and delayed intervention participants.

Characteristics		All (N=34)	Immediate intervention (n=17)	Delayed intervention (n=17)	*P* value^a^
Women, n (%)		28 (82)	14 (82)	14 (82)	N/A^b^
Age in years, mean (SD)		55.5 (8.6)	52.3 (9.7)	58.7 (6.0)	.03
**Marital status, n (%)**					.40
	Married or common law	25 (74)	11 (65)	14 (82)	
	Separated or divorced	5 (15)	4 (24)	1 (6)	
	Widowed, never married, or other	4 (12)	2 (12)	2 (12)	
**Gross annual household income in Can $, n (%)**					.52
	≤12,000	2 (6)	1 (6)	1 (6)	
	12,001-24,000	1 (3)	0	1 (6)	
	24,001-40,000	2 (6)	0	2 (12)	
	40,001-60,000	5 (15)	4 (24)	1 (6)	
	60,001-80,000	0	0	0	
	80,001-100,000	3 (9)	2 (12)	1 (6)	
	>100,000	14 (41)	7 (41)	7 (41)	
	No answer	7 (21)	3 (18)	4 (24)	
**OA^c^ diagnosis, n (%)**					.73
	Yes	20 (59)	11 (65)	9 (53)	
	No, but met the “likely OA” criteria	14 (41)	6 (35)	8 (47)	
**“In general, would you say your health is…”, n (%)**					.15
	Excellent	6 (18)	5 (29)	1 (6)	
	Very good	11 (32)	5 (29)	6 (35)	
	Good	13 (38)	4 (26)	9 (53)	
	Fair	4 (12)	3 (18)	1 (6)	
	Poor	0	0	0	
**“Compared with 1 year ago, how would you rate your health in general now?”, n (%)**					.25
	Much better	1 (3)	1 (6)	0	
	Somewhat better	1 (3)	0	1 (6)	
	About the same	27 (79)	15 (88)	12 (71)	
	Somewhat worse	5 (15)	1 (6)	4 (24)	
	Much worse	0	0	0	
Number of comorbid conditions, median (25th; 75th percentile)		2.5 (2.0; 4.0)	3.0 (2.0; 4.0)	2.0 (2.0; 3.0)	0.53
Body mass index in kg/m^2^, mean (SD)		27.2 (4.7)	29.1 (4.5)	25.4 (4.2)	0.02

^a^*P* values were based on exact chi-square tests for categorical variables (nonmissing data), and 2-sample *t* tests for continuous variables.

^b^N/A: not applicable.

^c^OA: osteoarthritis.

**Table 2:** Feasibility assessment.

Feasibility component	Criteria	All participants (N=34)	Immediate group (n=17)	Delayed group (n=17)
Recruitment rate	≥80% of eligible individuals agreeing to participate	74% (34/46 eligible)	N/A^a^	N/A
Dropout rate	≤10% of participants dropping out	0%	0%	0%
Adherence to intervention and assessment protocol	≥85% participants adhering to the study protocol	88% (30/34 enrolled)	88% (15/17 enrolled)	88% (15/17 enrolled)
Loss or malfunction of SenseWear	≤10% SenseWear loss or malfunction	0%	0%	0%

^a^N/A: not applicable.

### Sample Size and Data Analysis

With the resources available for the feasibility study, we aimed to recruit 30 participants within an 8-week period. We used descriptive statistics to summarize the feasibility variables and baseline variables of the 2 groups. We explored preliminary efficacy using intention-to-treat analysis. Q-Q plots were used to assess normality of the outcome variables. We conducted analysis of covariance to evaluate the effect of the group type (immediate vs delayed) on the outcome measures assessed at the end of 1 month (T1) and 2 months (T2), after adjusting for blocking and baseline (T0). We assessed 3 planned contrasts of changes in outcome measures over the measurement periods. The first contrast compared T0 to T1 between the 2 groups to determine whether the intervention was superior to the control. The second contrast compared T0 to T1 in the immediate group against T1 to T2 in the delayed group. The third contrast compared T0 to T1 in the immediate group against T0 to T2 in the delayed group. The last 2 models assessed whether the 1-month delay had an impact on the effect of the intervention.

We assessed the impact of missing data on the estimated effects of group assignment using imputation methods as described in van Buuren [45]. Specifically, we generated 10 imputed values using alternative random variates derived in a linear regression model, which included group, sex, baseline age, and baseline body mass index as predictors. We repeated the analyses using the 10 imputed values, and compared the conclusions and estimates against the main analysis, which assumed that data were missing at random.

### Ethics Approval

The research protocol was approved by the University of British Columbia Clinical Research Ethics Board (application number: H14-02631), was registered with ClinicalTials.gov (NCT02313506), and has been published in the peer-reviewed literature [26].

## Results

**Figure 1:**
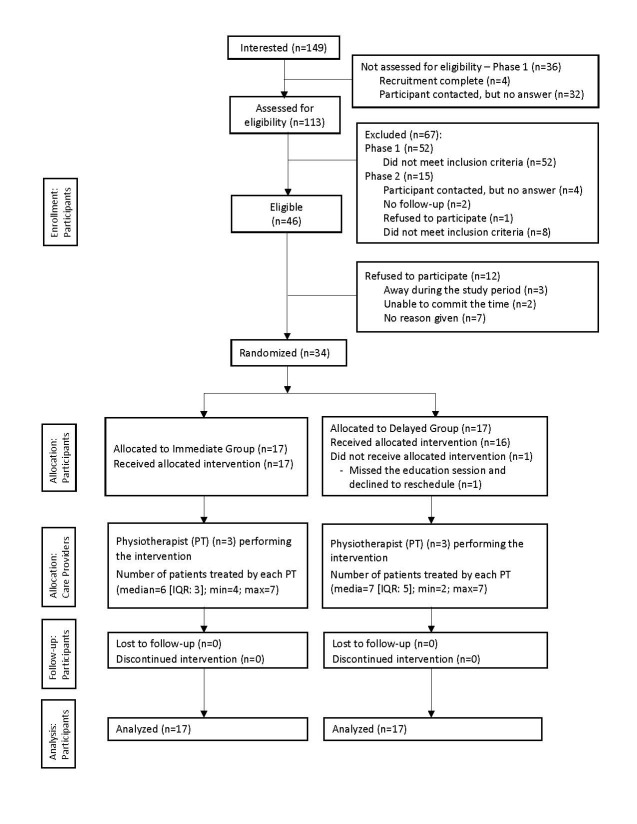
Consolidated Standards of Reporting Trials (CONSORT) flowchart. IQR: interquartile range.

Between January and March, 2015 (7 weeks), 149 people expressed an interest to participate, and 46 met the eligibility criteria. Of those, 34 were enrolled and completed the study (Figure 1). The majority of participants were women (n=28, 82%), with the immediate group (mean age 52.3, SD 9.7 years; n=17) younger than the delayed group (mean 58.7, SD 6.0 years; n=17). A total of 20 participants (59%) reported a diagnosis of OA, and 14 (41%) met the “likely OA” criteria without a diagnosis. Among the participants, 17 rated their health as “very good” or “excellent.” The mean body mass index was 27.2 (SD 4.7) kg/m^2^, with the immediate group (mean 29.1, SD 4.5) higher than the delayed group (mean 25.4, SD 4.2) (Table 1).

### Feasibility

Our recruitment strategy identified 46 eligible individuals; of those, 34 (74%) enrolled and none dropped out (Table 2). All but 1 participant adhered to the intervention protocol. All participants completed the assessments as per protocol at T0 and T1. Participants were required to wear a SenseWear (the primary outcome measure) for at least 4 days [46], with each day requiring less than 4 hours of off-body time to be included in the analyses. All 34 participants met these wear criteria at T0 (mean number of days worn: 5.9, SD 0.3; mean off-body time: 23.1, SD 13.6 minutes) and T1 (mean number of days worn: 5.6, SD 0.7; mean off-body time: 24.8, SD 18.0 minutes). At T2, 31 participants adhered to the wear criteria (mean number of days worn: 5.7, SD 0.6; mean off-body time: 38.4, SD 27.9 minutes). In the delayed group, 1 participant did not complete the outcome measures.

### Preliminary Efficacy

**Figure 2:**
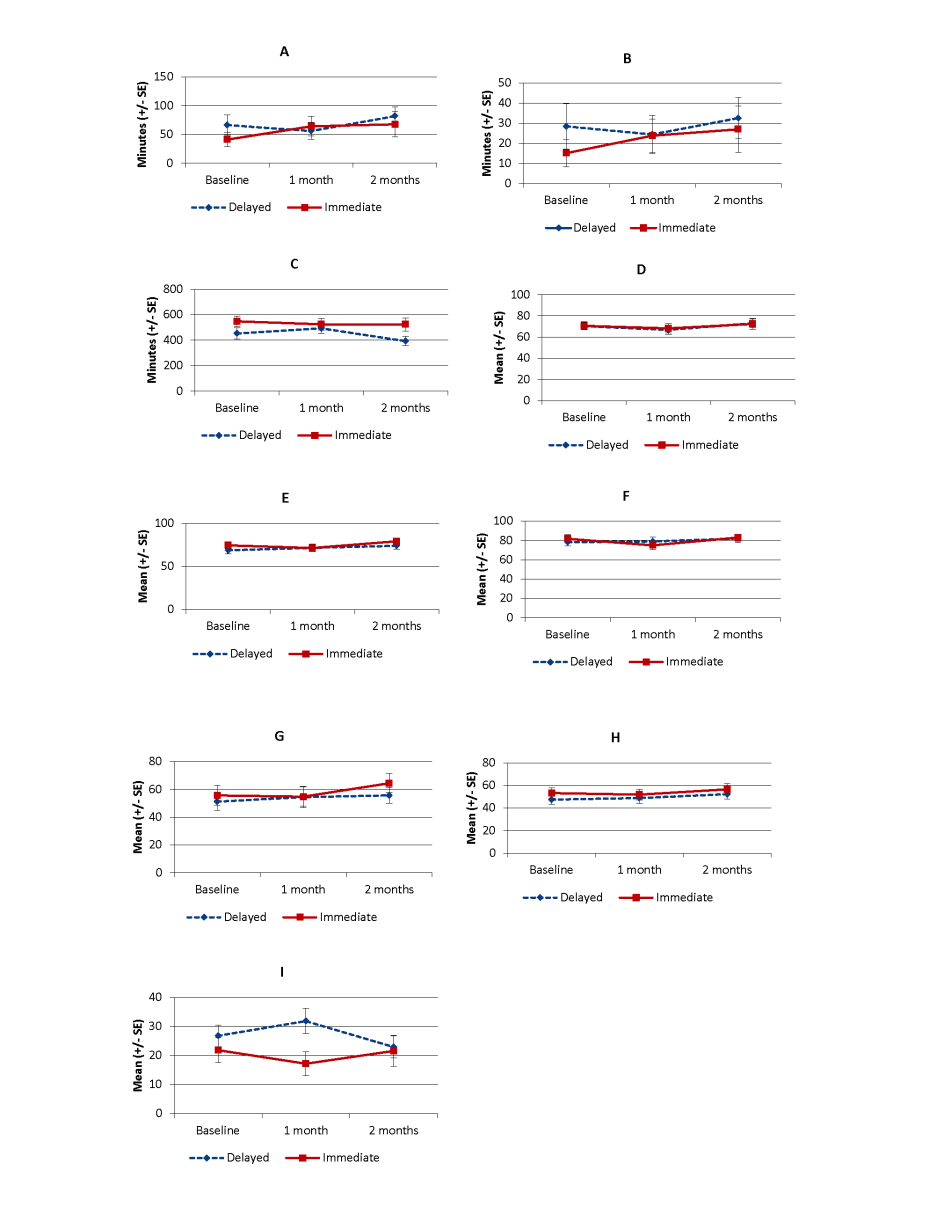
Results of outcome measures. (A) Bouted moderate to vigorous physical activity (≥3 metabolic equivalent tasks [METs]). (B) Bouted moderate to vigorous physical activity (≥4 METs). (C) Bouted sedentary time. (D) Knee Injury and Osteoarthritis Outcome Score (KOOS) symptoms subscale. (E) KOOS pain subscale. (F) KOOS activities of daily living subscale. (G) KOOS sports and recreation subscale. (H) KOOS quality of life subscale. (I) Partners in Health Scale.

Figure 2 shows the results of outcome measures from 3 time points. Prespecified contrast analyses revealed a significant effect whereby the immediate group improved in the MVPA (≥3 METs) time at T0 to T1 compared with the delayed group (contrast coefficient –31.1, 95% CI –56.6 to –5.7; *P*=.02) (Table 3). We also found a significant effect in the Partners in Health Scale scores at T0 to T1 (contrast coefficient 10.9, 95% CI 2.5-19.3; *P*=.02). The planned contrast of the immediate group at T0 to T1 versus the delayed group at T1 to T2 showed a significant effect in sedentary time (contrast coefficient –83.6, 95% CI –154.1 to –13.1; *P*=.03) and the KOOS symptoms subscale (contrast coefficient 6.9, 95% CI 0.4-13.5; *P*=.048), favoring the delayed group at T1 to T2. We found no significant effect in any outcome measures in the contrasts comparing the immediate group at T0 to T1 with delayed group at T0 to T2.

**Table 3:** Participant outcomes and results of contrast analyses.

Outcomes		Immediate intervention (n=17)			Delayed intervention (n=17)			Adjusted group effect immediate intervention vs delayed intervention coefficient (95% CI)^a^		
		Baseline	1 month	2 months	Baseline	1 month	2 months	Contrast 1	Contrast 2	Contrast 3
Daily bouted MVPA^b^: ≥3 METs^c^ (minutes), mean (SD)		41.3 (51.6)	64.2 (70.5)	67.7 (85.8)	66.5 (71.0)	56.0 (60.1)	81.9 (64.4)	–31.1 (–56.6 to –5.7)	4.6 (–19.6 to 28.9)	–11.0 (–31.1 to 9.1)
Daily bouted MVPA: ≥4 METs (minutes), mean (SD)		15.1 (27.9)	23.8 (34.0)	27.0 (44.6)	28.4 (46.3)	24.5 (39.0)	32.6 (40.5)	–10.5 (–23.4 to 2.4)	1.2 (–9.6 to 12.0)	–4.7 (–12.0 to 2.6)
Daily bouted sedentary time^d^ (minutes), mean (SD)		548.4 (169.1)	524.9 (192.1)	523.9 (200.2)	453.3 (180.5)	492.8 (164.8)	393.1 (144.2)	51.4 (–18.4 to 121.3)	–83.6 (–154.1 to –13.1)	–46.3 (–109.0 to 16.4)
**Knee Injury and Osteoarthritis Outcome Score^e^subscales, mean (SD)**										
	Symptoms	70.6 (15.8)	68.3 (18.4)	72.5 (20.2)	70.4 (14.9)	66.8 (18.2)	73.2 (15.4)	–1.3 (–7.5 to 5.0)	6.9 (0.4 to 13.5)	3.8 (–3.9 to 11.4)
	Pain	74.5 (16.2)	71.4 (17.5)	79.1 (13.0)	68.6 (16.1)	71.6 (15.2)	74.0 (15.4)	3.9 (–4.9 to 12.6)	3.4 (–4.3 to 11.1)	5.0 (–3.8 to 13.8)
	Activities of daily living	81.8 (17.1)	75.1 (19.7)	83.0 (14.9)	78.3 (15.9)	79.1 (18.9)	82.2 (17.1)	7.2 (–1.4 to 15.8)	7.8 (–1.2 to 16.9)	8.4 (–0.8 to 17.6)
	Sport and recreation function	55.6 (29.5)	54.7 (28.3)	64.4 (28.4)	51.2 (26.0)	54.4 (31.4)	55.6 (22.6)	3.6 (–8.2 to 15.4)	–0.5 (–11.0 to 10.0)	3.2 (–7.9 to 14.4)
	Knee-related quality of life	53.3 (18.4)	51.8 (19.5)	56.6 (20.2)	47.4 (16.1)	48.9 (19.3)	52.3 (18.0)	2.6 (–5.0 to 10.3)	3.7 (–3.0 to 10.4)	5.5 (–2.0 to 13.0)
Partners in Health^f^, mean (SD)		21.9 (17.6)	17.2 (17.0)	21.6 (21.9)	26.8 (15.3)	31.9 (17.9)	22.9 (15.2)	10.9 (2.5 to 19.3)	–0.3 (–10.3 to 9.7)	1.9 (–6.0 to 9.8)

^a^Outcome and baseline times are as follows: contrast 1: immediate intervention T0 to T1 vs delayed intervention T0 to– T1; contrast 2: immediate intervention T0 to T1 vs delayed intervention T1 to T2; contrast 3: immediate intervention T0 to T1 vs delayed intervention T0 to T2. Contrast models were adjusted for block sizes and baseline outcome measure.

^b^MVPA: moderate to vigorous physical activity, performed in bouts ≥10 minutes.

^c^METs: metabolic equivalent tasks.

^d^Sedentary behavior was performed in bouts >20 minutes.

^e^Scores range from 0 to 100, with higher being better.

^f^Scores range from 0 to 96, with lower being better.

Results from the imputation analysis (data not shown) were in line with the main missing-at-random analysis, in estimates, standard errors, and *P* values of group effects. This suggests that the presence of missing data did not have an important effect on the findings. No adverse event associated with the intervention was reported by participants during the study.

## Discussion

This study demonstrated the feasibility of a behavioral intervention, supported by the use of a wearable device, to promote physical activity. While our strategy yielded a recruitment rate below the goal of 80%, we exceeded the target in participant and equipment retention in a community-based study. Since our eligibility criteria were in line with other physical activity intervention studies involving people with knee OA [4], we will use the same eligibility criteria and plan sufficient time for participant recruitment in the future RCT. Furthermore, with 88% of participants adhering to the study protocol and no adverse events reported, we have shown that the intervention and study protocol can be delivered within the resource constraints.

Our results have also demonstrated preliminary efficacy of the physical activity counselling program, with the immediate group showing a trend of improvement in MVPA (≥3 METs) and the Partners in Health Scale compared with the delayed group at T0 to T1. Also, changes in MVPA appeared to be similar in both groups after they received the intervention. These findings are in line with previous studies on physical activity interventions, which generally result in short-term improvement (within 6 months) [4].

Results on sedentary behavior, however, were unexpected. While there was no noticeable effect at T0 to T1 between the 2 groups on these outcome measures, the intervention appeared to have a more favorable effect in the delayed group (T1 to T2) than in the immediate group (T0 to T1). One plausible explanation may be due to the counselling protocol. Although the program had separated physical activity and sedentary behavior into 2 counselling conversations, our approach might be more suitable for motivating people to be active than for encouraging them to sit less. We instructed the PTs to begin by asking participants about what they did to achieve the desired behavior (ie, being more active and sitting less) in a normal day. While this approach was logical for participants to set goals about their preferred physical activities, it might be less intuitive to think of ways to reduce sitting time, especially for those who had a sedentary occupation (eg, office workers or long-distance truck drivers). For them, focusing on what to do to reduce sitting highlighted the reality that participants had little control over this behavior, and therefore it might be challenging for them to set achievable goals. Similar issues have been raised by several recent systematic reviews on interventions to change activity behavior [47-49]. They concluded that, although programs targeting physical activity or combined activity and sedentary behavior are effective at improving physical activity participation, only the ones that are designed to change sedentary behavior achieve the best results in reducing sitting time. Given the challenge, it was possible that our study PTs needed time to practice and become comfortable with the sedentary counselling protocol. This might have contributed to the trend of improvement among participants who received the intervention later in the study. In light of the findings, we have refined the sedentary behavior counselling protocol with the study PTs and provided training sessions. The revised PT training protocol has been applied to 3 ongoing RCTs that are examining the efficacy of the program for patients with knee OA, rheumatoid arthritis, and systematic lupus erythematosus (ClinicalTrial.gov identifiers: NCT02315664; NCT02585323; NCT02554474). The first 2 registration numbers are for OA studies and the last number is for a study in people with rheumatoid arthritis and systemic lupus erythematosus.

Our result in the KOOS symptoms subscale was similar, with a significant difference found between the immediate group at T0 to T1 and the delayed group at T1 to T2. The reason for this is unclear, but it should be viewed in the context of the lack of a significant difference in other KOOS subscales, which are also associated with symptom severity. It should be noted that we did not adjust the analysis for multiple comparisons; hence, we cannot rule out the possibly that the results were due to chance.

This study has several limitations. First, we did not assess the full spectrum of feasibility. Although the study has identified strengths and areas of improvement for the intervention, we did not address *demand* (ie, intent to use) and *acceptability* (ie, intent to continue use and satisfaction) by people with arthritis [31]. Second, our sample was relatively active as indicated by the high bouted MVPA (≥3 METs) minutes at baseline. Since patients who are inactive are more likely to need active intervention, improvement in our recruitment strategy is needed to ensure that we reach this population in the full RCTs. Finally, 82% of participants were women. Since men and women may respond to behavioral interventions differently, additional efforts are required to enroll men in order to permit analyses to examine the effect of sex on the behavioral and disease-related outcomes.

These limitations notwithstanding, in the Track-OA study we have developed a physical activity counselling program that is practical and can be implemented in a full RCT. We have also demonstrated that it is feasible to use an objective physical activity measure (ie, SenseWear) for data collection in the community. The results have contributed to refining the counselling protocol, the recruitment strategy, and the timeline for a series of studies to evaluate the efficacy of this program.

## Appendix

Multimedia Appendix 1 Arthritis by physical activity. Data from Government of Canada, generated from: http://66.240.150.17:9600/PHAC/ readPPReports.jsp?l=en&folder=iDE8F756E1ED845419EE7457066BB23D5.

## Abbreviations

KOOS: Knee Injury and Osteoarthritis Outcome Score
METs: metabolic equivalent tasks
MVPA: moderate to vigorous physical activity
OA: osteoarthritis
PAR-Q: Physical Activity Readiness Questionnaire
PT: physical therapist
RCT: randomized controlled trial
