# Supplementary material for: A Community-Based Physical Activity Counselling Program for People With Knee Osteoarthritis: Feasibility and Preliminary Efficacy of the Track-OA Study
Source: JMIR Mhealth Uhealth. 2017 Jun 26;5(6):e86. doi: 10.2196/mhealth.7863 (PMC5504340; doi:10.2196/mhealth.7863)
Supplement: Multimedia Appendix 1 [file mhealth_v5i6e86_app1.pdf]

Arthritis by Physical Activity

Note: This data cube displays pre-calculated statistics. Only one selection must be made for each level.

Results

Percentage		Physically inactive
**Without arthritis**	**Percentage**	45.6
	**Lower 95% confidence limit**	45.1
	**Upper 95% confidence limit**	46.2
**With arthritis**	**Percentage**	57.2
	**Lower 95% confidence limit**	56.0
	**Upper 95% confidence limit**	58.4

Date modified:
2016-09-16
